# Comparing the Chemical Structure and Protein Content of ChEMBL, DrugBank, Human Metabolome Database and the Therapeutic Target Database

**DOI:** 10.1002/minf.201300103

**Published:** 2013-12-11

**Authors:** Christopher Southan, Markus Sitzmann, Sorel Muresan

**Affiliations:** [a] IUPHAR Database and Guide to PHARMACOLOGY web portal Group, The University/British Heart Foundation Centre for Cardiovascular Science, Queen’s Medical Research Institute, University of Edinburgh EdinburghEH164TJ, UK; [b] Chemical Biology Laboratory, Frederick National Laboratory for Cancer Research, National Cancer Institute, National Institutes of HealthFrederick, 21702 MD, USA; [c] Food Control Department, Banat’s University of Agricultural Sciences and Veterinary MedicineCalea Aradului 119, 300645 Timisoara, Romania

**Keywords:** Compounds, Proteins, Drugs, Drug targets, Databases, InChI

## Abstract

ChEMBL, DrugBank, Human Metabolome Database and the Therapeutic Target Database are resources of curated chemistry-to-protein relationships widely used in the chemogenomic arena. In this work we have extended an earlier analysis (PMID 22821596) by comparing chemistry and protein target content between 2010 and 2013. For the former, details are presented for overlaps and differences, statistics of stereochemistry as well as stereo representation and MW profiles between the four databases. For 2013 our results indicate quality improvements, major expansion, increased achiral structures and changes in MW distributions. An orthogonal comparison of chemical content with different sources inside PubChem highlights further interpretable differences. Expansion of protein content by UniProt IDs is also recorded for 2013 and Gene Ontology comparisons for human-only sets indicate differences. These emphasise the expanding complementarity of chemistry-to-protein relationships between sources, although different criteria are used for their capture.

## 1 Introduction

Databases that include explicit mappings between proteins and the small-molecules that interact with them as bioactivity modulators offer expanding opportunities in chemogenomics and pharmacological informatics. However, their proliferation also presents challenges. One of these is to discern incremental utility of individual resources and their combinations in various portals, for particular tasks. The interpretation of integrated results needs an understanding of each database from which they are extracted.[1] This is essential to judge between the inevitable noise and discordance in merged entities or result relationships. In addition, the reassurance engendered by apparent independent concordance can be confounded by the increasing circularity of data records (i.e. re-cycling of the same primary data between databases).

The key to assessing utility is to compare databases in detail and thereby acquire an understanding of the different rules by which they have been populated. This work outlines ways of approaching this by using four well-established and high-value databases: ChEMBL,[2] DrugBank,[3] Human Metabolome Database (HMDB),[4] and the Therapeutic Target Database (TTD).[5] We undertook a study of these four databases in 2010, although this was not published until 2012.[6] This new work extends our earlier study in two main ways. Firstly, all four resources have undergone major updates. We can thus now gain unique insights from comparing snapshots taken approximately four years apart. Secondly, developments such as wider adoption of the InChI, the inclusion of all four sources in PubChem, new cross-references in the UniProt database and additional cheminformatic options, have allowed us to expand the scope of the 2013 analysis. Since 2010 new methods for indexing molecules have been described, including an extended version of the Morgan algorithm, and compared with existing ones.[7] However, in the interests of comparative consistency between our two studies we have retained the main features of our previous analysis pipeline. Additional context to this work is provided by new publications that have since appeared from each database. Notwithstanding, brief summaries are provided below, along with self-reported entity counts from the release versions used in this work.

– ChEMBL data is mainly curated from journals covering a significant fraction of global medicinal chemistry reports and structure-activity-relationship (SAR) results. Release 15 (January 2013) specifies on the website: 9570 targets, 1254575 distinct compounds, 10509572 activities and 48 735 publications (n.b. release 16 appeared as this work was being finalised).
– DrugBank collates target and mechanism-of-action information. Version 3.0 (January 2011) contains 6715 drug entries including 1452 FDA-approved small molecules, 131 biologicals, 86 nutraceuticals and 5076 experimental compounds. These are mapped to 4233 protein IDs. Half the detailed information in the records is devoted to the drug, the other half to sequences, pharmacological properties, pharmacogenomic data, food-drug interactions, drug-drug interactions and experimental ADME data.
– HMDB collates detailed chemical, clinical and biochemical data on human metabolites. These are linked to other databases including enzymes involved in the transformations. Version 3.0 (September 2012) contains 40437 chemical entries and 5650 protein sequence identifiers. Because they have both been developed at the same institution, linkages are provided between DrugBank and HMDB at the compound, protein and pathway levels. In May 2013, HMDB switched their version number to 3.5, but without major changes in the data.
– TTD is conceptually similar to DrugBank but the compound-to-target mappings are focussed on primary targets. Another difference is the three-way split of targets and compounds into marketed, clinical trial and research phase. The latest version 4.3.02 (August 2011) includes 2025 targets, 17816 chemical structures, including 1540 approved drugs.

## 2 Methods

Our analysis is divided between the two main themes of chemistry and proteins. The following section will outline the basic steps and how these were enhanced in 2013 but for a full oversight we recommend consulting our previous publication.[6]

### 2.1 Chemistry Comparison

For the extrinsic analysis of chemistry we included the sets available for download as SD file from each website in September 2010 with those available in January 2013. The questions we wanted to answer are how the set of chemical structures and the number of unique structures in each of the four databases has grown, how much the older version (2010) overlaps with the newer one (2013), and also how the structural overlap between the databases has changed. Table 1 provides an overview of versions, structure record counts of the original files and a comparison to the number of current Substance records in PubChem (generated by PubChem Query “Database Name”[SourceName]). For all databases we found small variations in record counts between the downloadable 2013 SD files, the Substance (SID) count in PubChem and structure counts mentioned on the databases. In cases where these discrepancies were large we sought to provide an explanation.

**Table 1:** Versions and file downloads.

Database	Year	Version	Release date	Declared source record count	Substance (SID) count in PubChem (May 2013)
ChEMBL	2010	6	2010-09-02	600625	804093
	2013	15	2013-01-30	1251913	
DrugBank	2010	2.0	2008-01-31	4886	6683
	2013	3.0	2011-01-31	6516	
HMDB	2010	2.5	2010-09-19	7888	8550
	2013	3.0	2012-09-15	40209	
TTD	2010		2010-09-19	3616	14771
	2013		2011-08-25	15009	

In comparison to 2010, ChEMBL has more than doubled in the latest release (version 15, January 2013). The SID count for ChEMBL in PubChem is about 450 000 records smaller than the direct download from ChEMBL (see PubChem comparison section below). DrugBank has grown ∼30% between version 2.0 and 3.0 published in 2011. For HMDB, we used 2.5 in our 2010 study. The SD file from September 2010 contained 7888 records (although some failed processing) but when 2.5 was re-downloaded in early 2013 we found 8553 structure records. The latest HMDB (3.0) has grown to ∼40000 records in the download file. TTD increased between 2010 and 2013 to ∼15000 structure records and has a similar count of Substances in PubChem (14771 SIDs).

The content of all original database SD files were processed with the cheminformatics toolkit CACTVS.[8] The structure records were normalised to our standards on basis of the rule sets implemented for the NCI/CADD identifiers FICTS, FICuS and uuuuu.[9] These differ in their normalisation modes, i.e. they have varying levels of sensitivity to certain molecular and atomic features. They thus have different scopes of what is regarded as a chemically unique structure. In the first step of normalisation a unique representation of stereochemistry, charged resonance structures, miss-drawn functional groups, undefined hydrogen atoms, undefined charges, and incorrect valences, are all addressed. From this level, a numeric hash code representation is generated that uniquely represents the normalised structure and establishes the FICTS identifier of the input structure. In addition, for the FICuS identifier, a canonical tautomeric representation is created before the hash code is calculated. This means the FICuS identifier is able to link different tautomeric forms of the same chemical compound as they occur in different or even the same source databases. Finally, the uuuuu identifier also disregards counter ions, stereochemistry, isotopic labelling, and formal charges in comparison to the FICuS identifier. It is thus useful to find related forms of the same chemical compound which share the same basic connectivity and skeleton, irrespective of tautomers, stereoisomers, salt forms or charged species.

For comparisons, the IUPAC International Chemical Identifier (InChI) Standard InChIKey (version 1.04) were calculated for all original structure records.[10] For this analysis, we also calculated a second set of InChIKey for which the new InChI flags “KET” and “T13” were switched on. In the latest version of the InChI library these allow for a stricter handling of tautomerism compared to Standard InChIKey and may be incorporated into a forthcoming version. All chemical identifiers were stored and organized in a MySQL database for further analysis. We also maintained the original record IDs of the source database (e.g. DBxxxxx, HMDBxxxxx and CHEMBLxxxxx) including the release designations used in Table 1. For the set of normalized (unique) structures in the database we used CACTVS to calculate molecular weights and comparative statistics related to the quality of stereo information.

Unlike 2010, when TTD and HMDB were not yet present, all four databases are now specifically selectable as submission sources in PubChem. There are two caveats. Firstly, completion of the submission of all HMDB structures is still pending because of curation updates.[11] The second is that, as explained in ChEMBL v.10 release notes of June 2011, ChEMBL substantial increased the number of compounds by including PubChem confirmatory BioAssays with dose-response endpoints (e.g., IC50, Ki, or potency). As a consequence, while ChEMBL v.15 declares 1254575 structures for the direct download, the PubChem query “ChEMBL”[SourceName] retrieves 804 093 CIDs. Thus, approximately 450000 structures from PubChem were imported into ChEMBL v.15. Notwithstanding these caveats, we took advantage of the PubChem toolbox to perform various comparisons. These are not only different in the information they provide, but are also complementary to the analysis of direct downloads. The filters used for the intersections were a combination of the default PubChem settings (seen on the lower left of any query result page) and three set up for this work. One which needs some explanation is patent occurrence. This was the union (in size order) of SureChemOpen, Thomson Pharma, SCRIPDB and IBM as submitting PubChem sources of patent-extracted structures.

### 2.2 Proteins

Comparisons of protein content were performed using lists of UniProt protein identifiers derived from each source. For TTD these were parsed from a text dump of the records.[12] For DrugBank the external database and ID links for drug targets were retrieved from the download interface.[13] Since HMDB was undergoing a site upgrade the equivalent UniProt IDs were obtained directly (courtesy of Dr Craig Knox). Because ChEMBL now has direct links from UniProt the appropriate query was used to retrieve the ID list.[14] These links were first instigated for ChEMBL v.14 in November 2012 and may not have yet been synched to ChEMBL v.15. However, because of the convenience of being able to query and intersect these entries directly from the UniProt interface, we have used this for the 2013 protein set. The Venny web tool was used to generate Venn diagrams.[15] The version numbers and dates are given in Table 1 but for convenience we refer from this point on to the historical and contemporary sets as “2010” and “2013”, respectively.

## 3 Results for Chemistry

Table 2 lists the number of unique structure records in the four databases.

**Table 2:** Unique structure counts. These were determined by Standard InChIKey, FICTS, and InChIKey with tautomer flags set to “T13” and “KET”, FICuS, and uuuuu. They are presented for the “2010” and “2013” release of each database, together with, in brackets, the percentage of the original structure counts in Table 1.

Database (2010)	Version	Standard InChIKey (%)	FICTS (%)	Tauto. InChIKey (%)	FICuS (%)	uuuuu (%)
ChEMBL	6	599879	599862	592419	598625	558260
		(99.8)	(99.8)	(98.6)	(99.6)	(92.9)
DrugBank	2.0	4674	4675	4666	4665	4529
		(95.6)	(95.7)	(95.5)	(95.5)	(92.6)
HMDB	2.5	7877	7877	7849	7851	7488
		(99.8)	(99.8)	(99.5)	(99.5)	(94.9)
TTD	2010-09-19	2834	2857	2826	2833	2574
		(78.3)	(79.0)	(78.1)	(78.3)	(71.1)

Database (2013)	Version	Standard InChIKey (%)	FICTS (%)	Tauto. InChIKey (%)	FICuS (%)	uuuuu (%)
ChEMBL	15	1251500	1251500	1240676	1247717	1127013
		(99.9)	(99.9)	(99.1)	(99.7)	(90.1)
DrugBank	3.0	6379	6404	6362	6395	6198
		(97.9)	(98.2)	(97.6)	(98.1)	(95.1)
HMDB	3.0	40154	40196	40108	40094	39131
		(99.8)	(99.9)	(99.7)	(99.7)	(97.3)
TTD	2012-10-01	14111	14152	13974	14107	13306
		(94.0)	(94.2)	(93.1)	(93.9)	(88.6)

Because of their different input normalisation stringencies, each of the identifiers indicates an expected reduction in the number of unique structures compared to the original counts, with bracketed numbers giving the percentage of this value. The upper part of Table 2 is a repeat of our 2010 results but performed with enhanced 2013 processing. These include InChI 1.04 instead of 1.03 and a newer version of CACTVS (academic 3.410 version, February 2012). This improved reading capabilities and comparability to processing of the most recent database releases. Nevertheless, we recorded only minor variations between our 2010 results and the repeated calculation in the upper part of Table 2.

The comparison of the unique structure counts obtained by Standard InChIKey vs. FICTS (Table 2) shows similar results for all four databases and intermediate releases. This might seem unexpected as the Standard InChIKey already includes some basic handling of tautomerism, while the FICTS identifier does not normalise tautomers. However, we have seen similar behaviour in other cases. For each of the two releases of ChEMBL and HMDB we found only a small number of duplicates by Standard InChIKey and FICTS (0.1% or 0.2%, respectively), while for DrugBank the number of duplicates decreases from ∼5% to ∼2% between both database versions. A dramatic reduction can be seen for the 2010 version of TTD for which the number of unique structures is 20% lower than the original structure record count. The change to ∼6% in the 2013 version indicates an improvement in the quality of chemical structures in TTD.

If a stricter handling of tautomerism is performed via the FICuS identifier and the second set of calculated InChIKey (i.e. switching on the InChI tautomer flags “T13” and “KET”), the unique structure counts reduce further. However, the effect is small if compared to the numbers of FICTS and Standard InChIKey. Changes and improvements between 2010 and 2013 are very similar for all databases and their releases.

The uuuuu identifier offers a diversity assessment of basic connectivity (i.e. it disregards that “diversity” created by different stereoisomers, charged forms, salts as well as tautomeric forms). It also estimates the number of unique chemical skeletons (including bond orders). Thus, for ChEMBL, DrugBank, and HMDB, the uuuuu counts of unique structures are ∼6 and ∼12% lower than the original structure count, with the exception of the 2010 version of TTD that dropped ∼30%.

The impression given by Table 2 is that the database teams have reduced duplicates and improved their handling of different tautomeric forms of the same canonical compound. Note that from the data per se if a database both expands and improves on average, we cannot discriminate between remediation of existing structures, marked improvements of just the new ones, or both.

**Figure 1:**
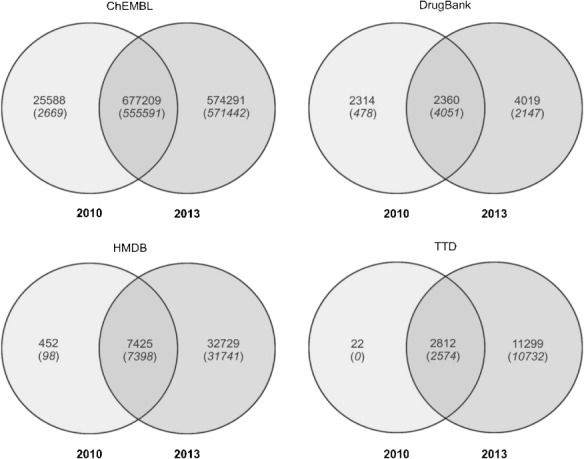
Venn comparisons for the 2010 and 2013 versions. The main numbers are overlap by Standard InChIKey, those underneath in italics and brackets are by uuuuu.

Our discussion of content overlap will be restricted mainly to Standard InChIKey since this is the most established identifier. However, analysis by uuuuu also reveals interesting aspects. Figure 1 illustrates the individual changes between 2010 and 2013. The union sets of the Venn diagrams give the number of structures that have been maintained in both versions of each database, while the counts outside the union sets show the number of removed and newly added unique structures, respectively.

The much smaller number of unique structures reported by the uuuuu identifier can be explained in most cases by the different chemical scopes of uuuuu vs. Standard InChIKey identifier. For instance, for the 2010 release of ChEMBL we record 2669 unique structures by uuuuu compared to 25588 uniques by Standard InChIKey (Figure 1). This occurs mainly because of two effects. Firstly, the 25588 unique structures found by Standard InChIKey possess a low diversity. This means they form a much smaller set of unique structures when compared on basis of their basic connectivity, since this is what the uuuuu identifier is intended to do by disregarding stereoisomers, counterions, etc. Secondly, there are other cases where the uuuuu identifier subsumes structure records from the set exclusive to a single release into the union set of both releases. This occurs for records where the basic connectivity does not change between old and new database releases but improvements have been incorporated on some level in the newer release (e.g. adding or correcting stereochemistry). For these, the linkage between “original” and “improved” structure can only be established by disregarding the improvements. This is particularly the case for the union set of HMDB in Figure 1 where the count of unique structures by uuuuu is larger than by Standard InChIKey (4051 vs. 2360). Thus, these numbers also imply improved curation.

Table 3 shows the pairwise overlaps between the databases using Standard InChIKey for the database versions analysed in 2010 (upper part) and 2013 (lower part).

**Table 3:** Overlap matrix for the four databases by Standard InChIKey.

(2010)	ChEMBL	DrugBank	HMDB	TTD
ChEMBL	599879	1746	901	1622
DrugBank		4674	362	1220
HMDB			7877	163
TTD				2834

(2013)	ChEMBL	DrugBank	HMDB	TTD
ChEMBL	1251500	3335	4254	12893
DrugBank		6379	1746	1346
HMDB			40154	1306
TTD				14111

Unsurprisingly, since they all expanded, the overlaps between databases have increased in absolute numbers (the numbers in the main diagonal indicate the number of unique records by Standard InChIKey). For 2013 ChEMBL covers now substantially larger parts of DrugBank (up from 37% to 55%), and TTD (up from 57% to 91%) although TTD has grown itself in absolute numbers from 2834 to 14111 structure records (unique by Standard InChIKey).

**Figure 2:**
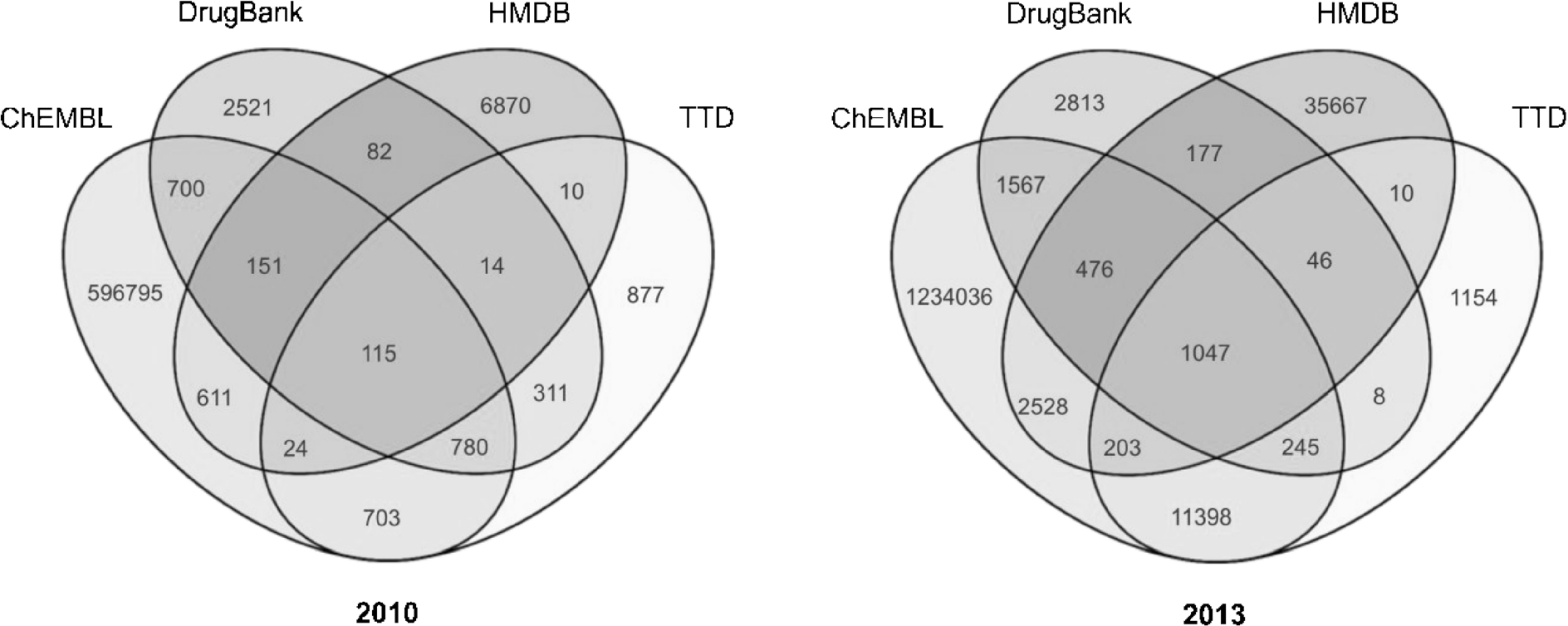
4-way Venn diagram of 2010 vs. 2013 structures

Figure 2 confirms this. The number of exclusive structures in TTD increases only moderately. However, the exclusive, mutual overlap with ChEMBL increases substantially (703 to 11398 unique structures). The new structures in HMBD seem to be largely unique content (see Table 3 and Figure 2) as expected considering HMDB’s focus on metabolites. DrugBank’s unique content does not change substantially, but has a much bigger overlap with ChEMBL. Given that ChEMBL is by far the largest database (and effectively doubled to 1.2 million), it is neither surprising that most of its content is unique relative to the other three databases nor that the overlaps concomitantly increase.

The set of structures in common (the centre union set in the Venn diagrams of Figure 2) increased from 115 to 1047 structures between 2010 and 2013. In 2010, we were surprised to record such low intersects since three of the databases should have included the same set of FDA-approved drugs. However, similar low intersects have been noted in an earlier comparison of drug databases.[1] In 2013, the 4-way union set (1047 structures unique by Standard InChIKey and 1270 by uuuuu) is closer to what three of the databases report as the numbers of approved drugs in their structure sets (TTD: 1540, DrugBank: 1424, and ChEMBL: 1214) but it should be noted that, via the inclusion of HMDB, this includes both drugs and metabolites.

A distribution of stereochemistry and stereo representation is provided in Table 4. From our experience, all other factors being equal, these parameters are indirect indicators of improved structure quality because the correct representation of stereochemistry requires a careful handling. However, the absence of stereo, or even seemingly incorrect representations may accurately represent what was specified in the extracted source, typically as an image or an IUPAC name (e.g. different journal papers referring to the same canonical structure). The statistics of this cannot therefore be used to assess curatorial accuracy.

**Table 4:** Analysis of stereochemistry between the “2010” and “2013” versions of the databases. The columns are (left to right) number of structures with no chiral atom stereo centers, structures for which stereo configuration of all atoms is specified, at least one stereo atom center is unspecified, those without bond stereo centers, those for which the configuration of all bond stereo centers is given, and those where at least one bond stereo center is missing. The percentage of the original structure count in Table 1 is given in brackets.

Database (2010)	No Atom Stereo Centres (%)	Full Atom Stereo Specification (%)	Unspecified Atom Stereo Specification (%)	No Bond Stereo Centres (%)	Full Bond Stereo Specification (%)	Unspecified Bond Stereo Specification (%)
ChEMBL	284553	148848	165944	530812	69632	176
	(47.4)	(24.8)	(27.6)	(88.4)	(11.6)	(<0.1)
DrugBank	1550	772	2542	4480	360	36
	(31.8)	(15.8)	(52.1)	(91.9)	(7.4)	(<0.1)
HMDB	1042	4763	2046	3051	4824	11
	(13.2)	(60.4)	(25.9)	(38.7)	(61.2)	(<0.1)
TTD	1583	1136	868	3223	388	5
	(43.8)	(31.4)	(2.4)	(89.1)	(10.7)	(<0.1)

Database (2013)						
ChEMBL	706502	222802	320428	1103833	128528	19517
	(56.4)	(17.8)	(25.6)	(88.2)	(10.3)	(1.6)
DrugBank	2622	2848	767	6018	469	29
	(40.2)	(43.7)	(11.8)	(92.4)	(7.2)	(<0.1)
HMDB	7547	6161	26 453	15 098	24 964	147
	(18.8)	(15.3)	(65.8)	(37.5)	(62.1)	(<0.1)
TTD	7826	4026	2886	13471	1311	8
	(52.9)	(27.2)	(19.5)	(91.1)	(8.9)	(<0.1)

Table 4 list the number of structures (plus the percentage of the original record count) without chiral atom centres, fully specified stereo configuration on all atoms, and undefined stereo configuration on at least one chiral atom. The situation for bond stereochemistry is given by the following metrics: a) the number of structures with no stereogenic double bonds, b) those that have correctly specified stereo configuration on all double bonds, and c) those with at least one double bond for which stereo information is missing.

Notably, the percentage of achiral structures has increased in all four databases between 2010 and 2013. We suggest this is due to expansion beyond drug-like compounds. The absolute numbers with full stereo specification has also increased but, because of overall growth, the relative numbers have fallen. Nevertheless, our stereochemistry results also imply enhanced curation efforts. For example, in DrugBank the number of structures with undefined atom stereo configuration dropped from 52% in 2010 to 12% in 2013. This is also recorded in the Venn diagram in Figure 1. For HMDB we recorded the opposite trend, where unspecified stereo information jumps from ∼25% to ∼65%. The 33000 new structures in 2013 thus included many with undefined stereo information. This may be associated with the increase in large lipids. HMDB has a much lower percentage of structures that are achiral in both the 2010 and 2013 versions. For bond stereo information it is striking that ∼90% of structures in the three drug-focused databases have no stereogenic double bonds. HMDB is the exception (37.5%) because of the focus on metabolites. The remaining two columns regarding bond stero information in Table 4 are difficult to interpret because double bonds are often arbitrarily drawn in E-configuration even though the actual stereo configuration is unknown.

**Figure 3:**
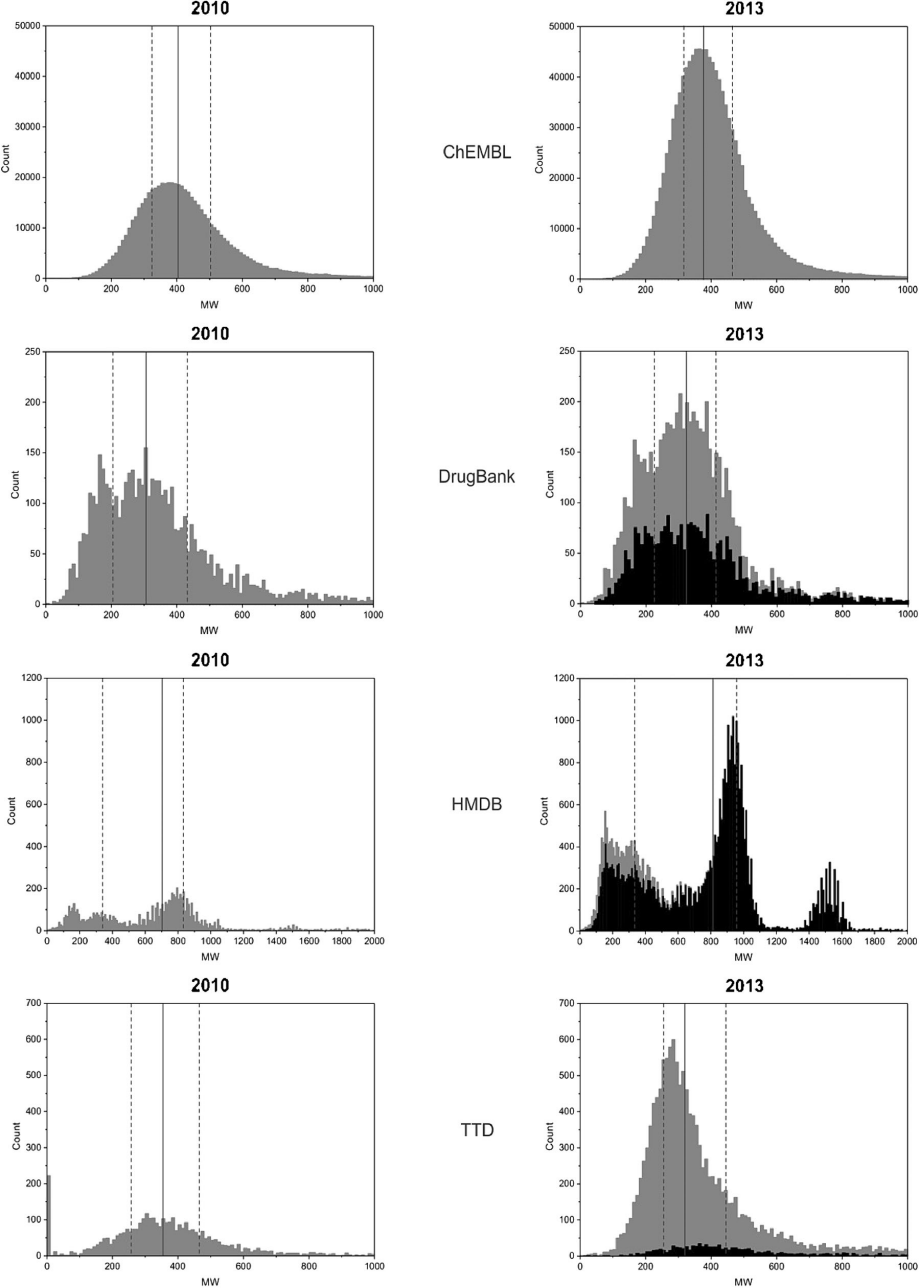
Molecular weight distribution of the 2010 and 2013 versions of ChEMBL, DrugBank, HMDB, and TTD. For 2013 the MW distribution of the exclusive structure records (i.e. exclusive content in Figure 2: 2521 records for DrugBank, 6870 records for HMDB, 877 records for TTD) is highlighted in black (for ChEMBL the grey and black distributions are basically identical). The solid line in each plot indicates the median, dashed lines represent Q1 and Q3, respectively. The statistics of these distributions are shown in Table 5.

While a series of chemical property profiling such as Log*P* and Polar Surface Area (*PSA*) would be of interest we had to restrict ourselves to *MW* as the most comparatively informative. This highlighted distinct differences (Figure 3 and Table 3, 5).

**Table 5:** Statistics for MW distributions displayed in Figure 3. The mean, median, standard deviation, lower (Q1) and upper (Q3) quartiles are shown.

	Mean	Std. Dev	Q1	Median	Q3
ChEMBL 2010	456	297	324	405	503
ChEMBL 2013	424	245	319	387	466
DrugBank 2010	364	303	205	309	428
DrugBank 2013	345	198	228	321	412
HMDB 2010	662	404	340	702	831
HMDB 2013	725	408	346	812	956
TTD 2010	400	366	258	354	466
TTD 2013	437	407	255	320	446

Starting with ChEMBL we can see a continuous distribution with the median ∼400. The implication is of a more “lead-like” than “drug-like” content.[16] This would fit with what might be expected from the extraction of SAR from the medicinal chemistry literature where the primary mode of activity testing is in-vitro. The 2010 distribution indicated the high-MW content had (proportionally) dropped slightly. The much smaller DrugBank collection shows a discontinuous spiky distribution but the median MW drops by ∼100 Da into a more “drug-like” zone compared to ChEMBL. While there is a hint of bimodality for 2010 this has smoothed out by 2013 with a slight rise in the median. This fits with the inference that these are predominantly in vivo optimised compounds and/or PDB ligands with concomitant lower average *MW* compared to ChEMBL. The same effect would be predicted for TTD and this is observed. However, the median is slightly higher from a larger proportion of high-*MW* entries compared to DrugBank.

The cumulative statistics of HMDB are confounded because of the pronounced tri-modal 2013 distribution. This is not only a major change but also indicates two large new clusters around ∼1000 and ∼1500. These are outside the envelope we might have expected for small-molecule metabolites as seen in the 2010 pattern. Inspecting selected entries under these peaks provided an explanation. For example, the first peak included a long-chain monolignoceric acid triglyceride with a MW of 1027 (HMDB47321). Under the second peak we find a cardiolipin of *MW* 1526 (HMDB57781). As a corroborative cross-check, the search terms “triglyceride” and “cardiolipin” retrieve 13923 and 3277 results, respectively, which approximately fits the peak size ratio and supports a focus on large lipid capture for the latest update.

**Figure 4:**
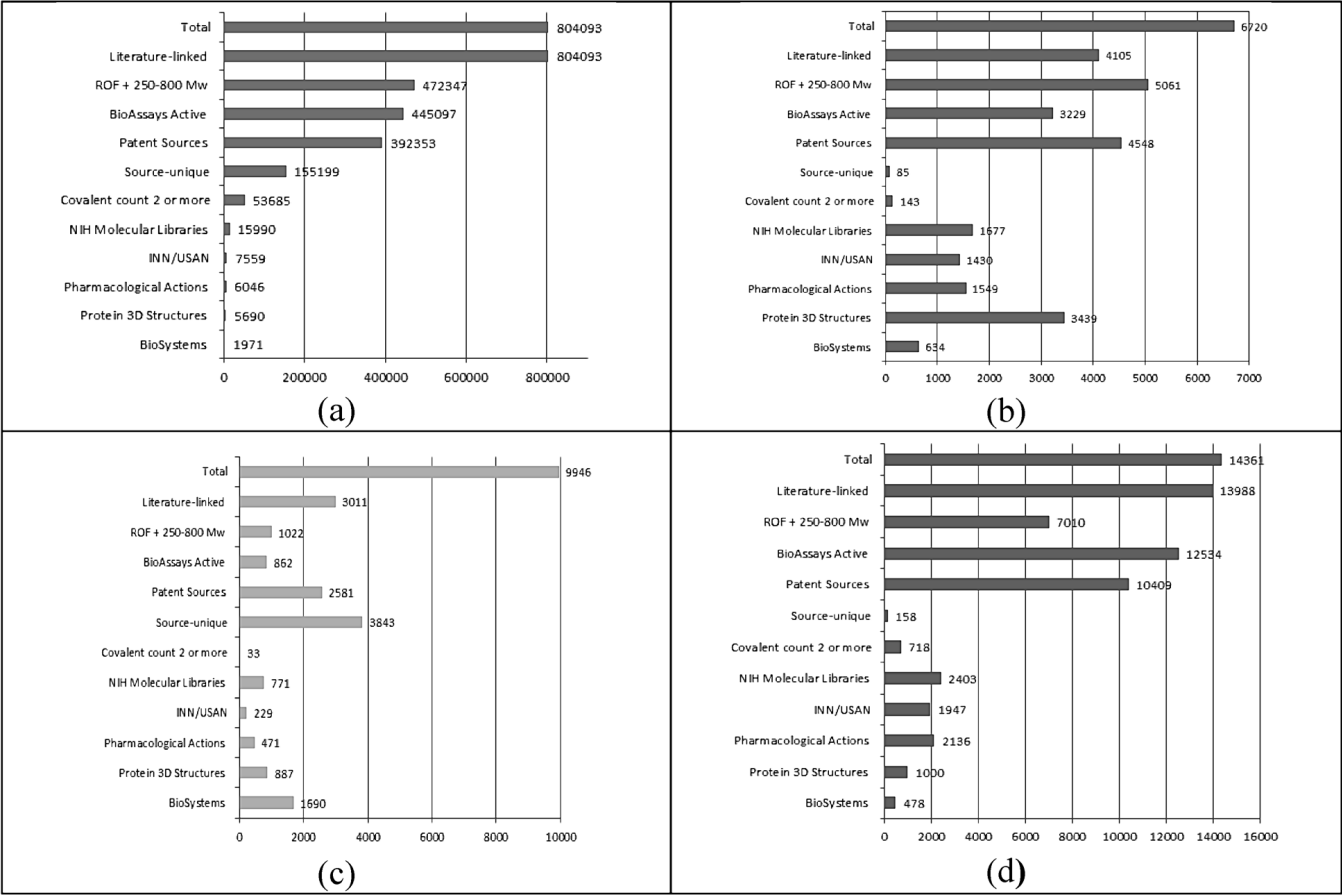
CID matches for selected sources in PubChem. The panels are: (a) ChEMBL, (b) DrugBank, (c) HMDB, and (d) TTD. The ranking of matches in ChEMBL was taken as the reference order for the other three plots.

This chemistry comparison section concludes with the results from comparing the databases inside PubChem where they are now instantiated as separate sources. We chose 11 categories of content to display in Figure 4. We can compare the 11 intersects for each database according to the order in Figure 4. The numbers in brackets after each category are the total CID counts for that filter in PubChem for May 2013.

*1. Literature-linked (962666).* Because this is largely ChEMBL-plus-PubMed the former is obviously recorded as 100%. The 60% coverage in DrugBank indicates lower primary literature capture but note these could be secondary sources such as review articles. It does raise the question as to which structures encompassed in the DrugBank 40% without primary literature but these may be PDB entries for which the individual reports are not cited. At 30% HMDB has the lowest literature coverage. An explanation is that the large number of lipid records can neither be cross-referenced to ChEMBL journal articles nor alternative PubMed IDs linked to a CID for the structure. In contrast to DrugBank, the proportion in TTD is up to 90%. This is corroborated by a 90% overlap at the structure level (Figure 3). The most likely explanation here is that the recent TTD curation efforts have actively selected ChEMBL entries as starting points and/or cross-links, whereas these only reach 60% in DrugBank.

*2. ROF+250–800 (3 1812 051).* This is a simple lead-like filter, encompassing drug-likeness at the lower MW end. While not predictive per se, this filter enriches for bioactive compounds (n.b. PubChem overall is skewed upwards in relative proportion because 75% of all vendor depositions are this range). Note that Figure 3 assists in the interpretation of the MW dimension. We can see that ChEMBL and DrugBank are similar at 58% and 61%, respectively. Lowest in this filter is HMDB at 10% but this is not unexpected considering many metabolites would fall below MW 250 and the substantial lipid content would be excluded on the basis of both LogP and MW. The data do not indicate any particular reason why TTD (49%) should be lower than both ChEMBL and DrugBank.

*3. Active in PubChem Bioassay (883627)*. For ChEMBL the figure of 55% seems unexpectedly low since this source is the major contributor to BioAssay, as mostly positive activity results of one sort or other extracted from the literature. However, ChEMBL data in PubChem does not get an active/inactive tag generated via thresholds in the same way as is done for the Molecular Libraries Screening Centre Network (MLSCN) result sets. The fact that this drops to 48% for DrugBank where one might expect positive linked assay results for all drug candidates may be related to the same active flagging issue. The 9% level of actives in HMDB is actually higher than anticipated since metabolites are not expected to be inhibitors at concentrations typically tested in assays but this is partially explained by drug content. The fact that TTD (87%) ranks significantly above ChEMBL in this filter also supports the idea that curation was specifically picking up compounds with potency data from ChEMBL.

*4. Patent Sources (15 039 047).* The observation that ∼50% of ChEMBL structures are in patent sources, compared to 13% overall in PubChem, can be explained by structures from the medicinal chemistry literature being first exemplified in patents. This rises to 67% and 71% for DrugBank and TTD respectively reflecting higher (proportional) drug and clinical candidate content. While TTD is low (26%) we would not expect metabolites to be claimed as structures. What may contribute to this are not only the drugs but also biochemical names in the dictionaries used for manual or automated patent extraction.

*5. Source-Unique (25806124).* This means the CID is specifically only from one submitter. At ∼20% the unique content of ChEMBL is the highest in the set, however, it would be even ∼100 K CIDs higher without the circularity arising from common chemical content between BindingDB and ChEMBL (i.e. the intersect between them represents 94% of the former). The interpretation here is that many of the structures extracted from papers by ChEMBL are (by CID rules) not hitherto represented in other PubChem sources. The caveats are not only that alternative stereo and/or other tautomeric representations may be present (i.e. single-source CIDs are not all canonically unique) but that any may be “correct” in reflecting representations derived from different extracted papers. As an example, the CID for CHEMBL1797692 (CID: 56680063, RAERAPYSCWYQAO-AKIFATBCSA-N) has fully specified stereo but the InChIKey skeleton matches a “flat” supplier compound (CID: 71369165, RAERAPYSCWYQAO-UHFFFAOYSA-N). The unique content of DrugBank and TTD is very low. The implication is that their structures are independently corroborated by other submissions merged in the CID records (e.g. ChEMBL as indicated above). However, this would be confounded if a proportion of curation was circular (i.e. did not involve *de novo* SID generation). At 38% the unique content of HMDB is high but 90% of this has a MW of above 800. Ranking these by MW and visual inspection indicates the unique content is substantially derived from complex lipids. This corroborates the MW results but note that ∼75% of the HMDB structures are not yet captured in this PubChem analysis. Corroborative information was supplied by an intersect with LipidMaps at 3576 (i.e. 35% inside PubChem).

*6. Disconnected structures of 2 or more components (1571436)*. This is a useful measurement for salts and mixtures (note this cannot technically discriminate dimers or other multimers but inspection suggests the occurrence of these is low). Only ChEMBL has a significant count (6.5%) which includes salt forms of drugs specified in the literature and USAN approvals.

*7. NIH Molecular Libraries (397823).* This is the physical collection shared between US screening centres. These structures can accumulate extensive cross-reactivity data depending on how long they have been in the collection. On a proportional basis the sources here do not have large intersects but ChEMBL is highest on an absolute basis. DrugBank is up to 25% which may be related to the high coverage of drugs and candidates.

*8. INN/USAN (10388).* Using this as an “and/or” query with a restriction to the PubChem Compound synonym field returns both currently approved drugs and historical advanced-stage candidates. It thus constitutes a comprehensive drug collection that is nominally independent (i.e. the above databases are not usually the first INN or USAN synonym-assigning sources). With a 72% intersect (with the total) ChEMBL has the largest coverage while both DrugBank and TTD are surprisingly low at 14% and 19% respectively. Possible explanations include differences in stereo and or salt forms. The databases may also have a lag time for newly approved drugs but there is no PubChem source from which these can be cleanly selected for comparison. The fact that HMDB includes a selection of drugs (229) is mentioned in their 2013 paper but note that some metabolites, vitamins and hormones also have INNs or USANs for pharmaceutical formulations.

*9. Pharmacological Actions (11 912.)* This important subset flags up where *in vivo* activity is assigned to a structure via MeSH curation of one or more PubMed IDs. It therefore indicates therapeutic testing of drug action in animal models and clinical trials with useful specificity. Here again ChEMBL scores high compared to DrugBank and TTD (at 51%, 13% and 18% respectively). Contributing factors here are the much larger scale of ChEMBL and the fact that many of the research compounds captured in DrugBank and TTD do not have *in vivo* characterisation data. The low figure in HMDB would include the drug and hormone content.

*10. Protein 3D Structures (23 562)*. This category indicates CIDs identified within a protein structure. The inclusion of all hetero-atoms exceeds specifically pocket-bound small-molecule ligands but intersecting these with the filter above (ROF+250–800) indicates ∼7900 could be in this category. While ChEMBL ranks top in absolute numbers (5690), DrugBank is proportionally highest (51%), significantly exceeding TTD (7%). This is in accord with the historical focus on ligands by DrugBank but this includes a small proportion of false-positives. An example is Alpha-D-Mannose (DB02944) classified as an experimental drug and mapped to 76 targets. The mapping is via hetero-atom entries rather than authentic ligands, although these proteins are not flagged with pharmacological action.

*11. Biosystems (9757).* This NCBI resource maps compounds into pathways via protein target links in BioAssay records and pathway databases.[17] Because of the many BioAssay target links in ChEMBL, the mapping is available for 20% of records there. In DrugBank and TTD the percentage is much lower with 6.5% and 4.9%, respectively. In HMDB, this information is available for 17% of all records which seems to be a quite high number, especially if factored by size relative to ChEMBL. However, by definition, many of the compounds are mapped into metabolic pathways.

## 4 Results for Proteins

### 4.1 2010 vs. 2013

**Figure 5:**
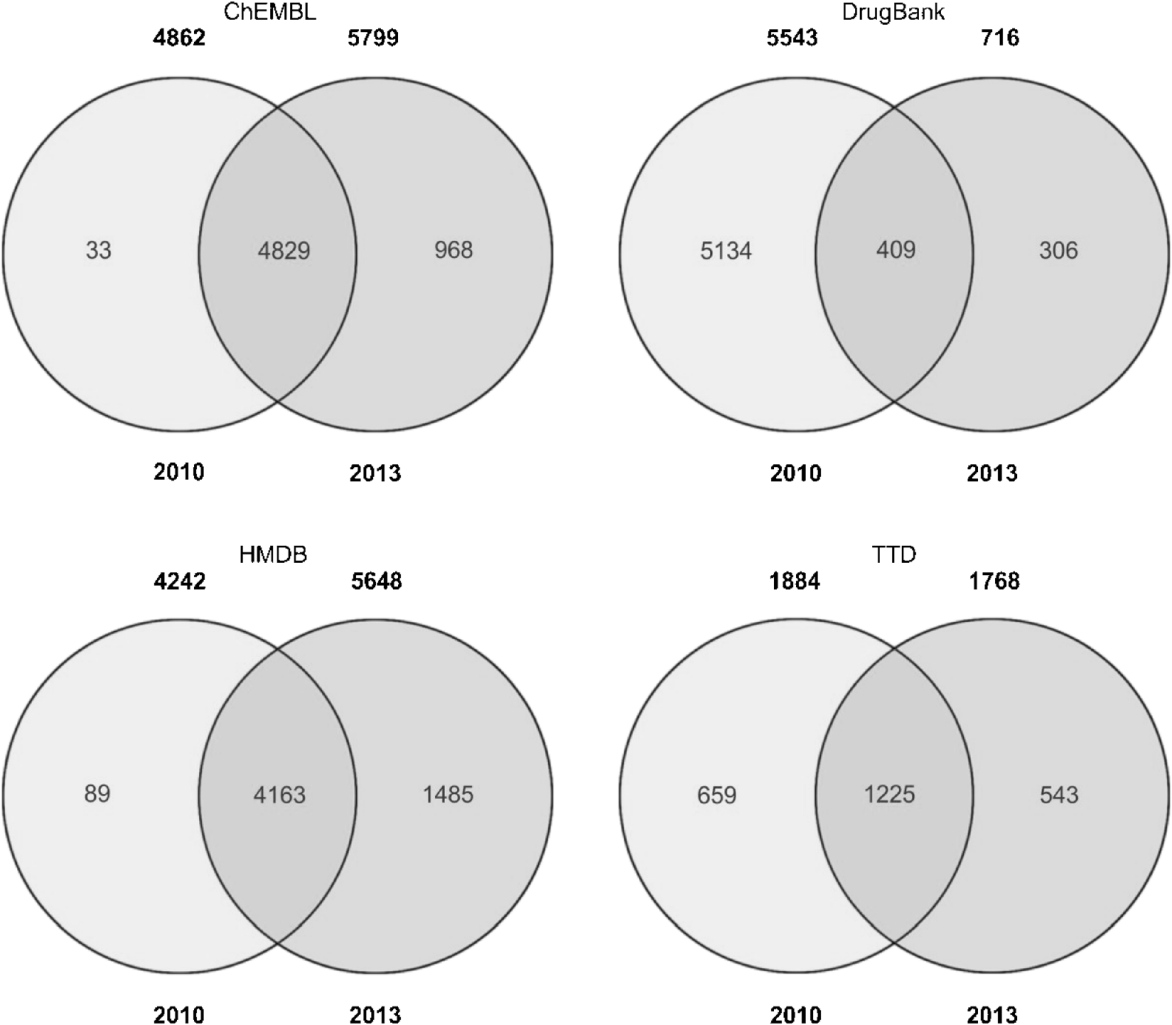
Protein content changes 2010 vs. 2013. The totals of the protein content for the two years are on top of each circle in the Venn diagram.

We recoded protein content changes in all four databases. We improved the resolution of identifiers in 2013 by accessing UniProt IDs directly, rather than use the Protein Identifier Cross-Reference Service (PICR) to BLAST-map downloaded FASTA sequences as we had utilised in 2010.[18] In addition, new DrugBank subsets have become available and HMDB content is being updated at the time of writing (May 2013). None of these changes are problematic per se since curation processes and underlying database schema are being continuously enhanced. Consequently, maintaining retro-consistency is impractical. However, this does make interpreting differences between old vs. new protein sets less valid than the analogous chemistry comparisons. This section will therefore focus more on comparing between the 2013 protein sets rather than old-vs-new. Nevertheless, we can start with 2010 vs. 2013 (Figure 5).

The results fall into two groups that we can term replacement and expansion. Thus, ChEMBL and HMDB have predominantly expanded whereas the other two have undergone removals as well as additions. The major change for DrugBank is due to the recent option of being able to download just those proteins that are flagged “Pharmacological action: yes” (highlighted in green in the Target records). This approximates to what could be termed a primary target mapping for the 716 proteins. While we could have chosen to compare different download sets from the same year, this would have made the analysis overly complex. Where a new choice is available, our default is to take the set with highest mapping specificity, even if this confounds retrospective comparisons. In Figure 5 the TTD 2013 set has also decreased but some of this may be due to discrepancies between our 2010 PICR cross-mapping and the 2013 direct ID download.

**Figure 6:**
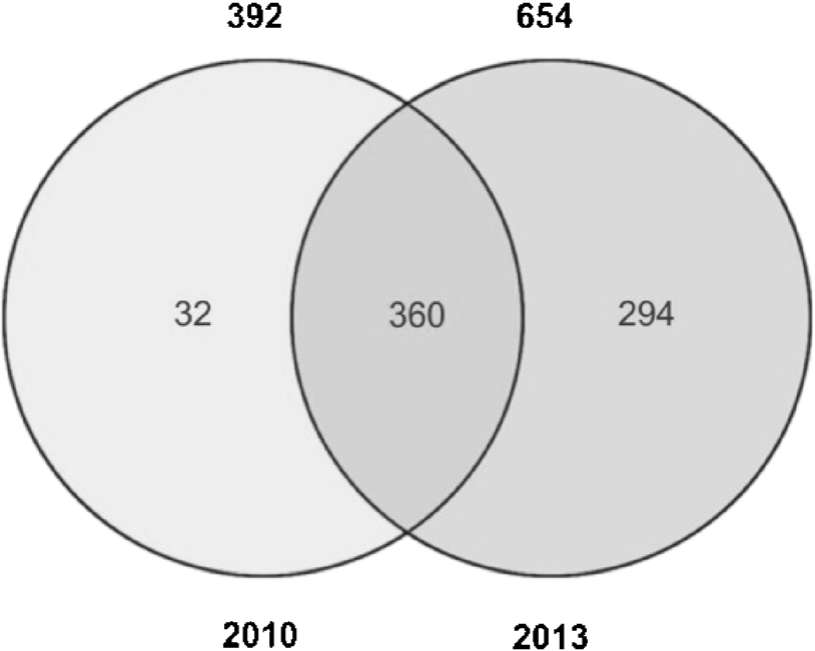
Comparison of the 2010 and 2013 target protein consensus intersects between ChEMBL, DrugBank, and TTD. The consensus totals are shown above the circles. For HMDB no target protein information is available.

Before moving on to the current proteins we performed a three-way comparison between just the three drug discovery databases between 2010 and 2013 because HMDB is “odd-man-out” in not sharing a similar target mapping concept (Figure 6). The comparison of database consensi in Figure 6 should be more robust than individual sets. The time point results have three features. The first is that target protein capture expanded by 60%. Secondly, when examined by the Panther classification[19] no significant shifts are detected (e.g. the receptor: enzyme ratio is similar despite expansion). The third feature is that all 32 proteins “lost” from the 2010 consensus are from DrugBank. The reason is that the smaller set of primary targets, selectable in 2013, has eliminated these 32 mappings. One of these, human serum albumin, provides particular classification challenges. In ChEMBL, P02768 is target-mapped to 650 compounds (as CHEMBL3253) with most of the 239 entries described as small-molecule binding assays because there is no “carrier” option. DrugBank has three unique entries for this protein as a biopharmaceutical, but also maps 104 small-molecules to P02768, classified as a “carrier” relationship, however, as expected, none have a “pharmacological action”. A different classification is used by TTD in mapping five small-molecules (including the antacid bismuth), to albumin (TTDS00336) but shows limited curatorial discrimination in assigning the default relationship of “successful target” (presumably because the five compounds were approved), in spite of the references clearly specifying the carrier function (except for the inclusion of one imaging agent). To complete the inter-database albumin survey, HMDB also maps the protein to nine small molecules, including several hormones (arguably also as a carrier), but classifies the protein type as “unknown” (HMDBP020759).

### 4.2 2013 Protein Comparisons

We initially addressed some basic questions. The first was to examine correlations between the statistics reported by the databases and our ID downloads. For ChEMBL the targets to UniProt ID ratio was 9570:5797 because not all targets can be protein-mapped). For instance, whole-cell screening is widely used to measure the growth inhibition effects for anti-infectives of all types in the primary literature. Consequently, searching ChEMBL with “plasmodium” gives 14 results and “mycobacterium” gives 38 results. Over 20000 compounds are thus mapped to these organisms as species “targets”, a relationship type not typically captured by the two drug databases. The latest DrugBank reported statistics are 4167 targets, 221 enzymes, 11 carriers (including albumin) and 120 transporters. While we recorded 4023 UniProt IDs from the data extractor download we selected the new primary target subset of 715. Currently, there are no reported protein content statistics for HMDB that we can compare with our download results but the mappings are under revision. The subsets from TTD specify 2025 “targets”, including 364 successful, 286 clinical trials, 44 discontinued and 1331 research. Our analysis found 1768 de-duplicated UniProt IDs Thus, for TTD “target” does also not always equate to a UniProt ID. For example, TDC00233 specifies “Gamma secretase” as a clinical trial target for two compounds. However, there is evidence of mixed curatorial rules because while this target entry was mapped to a UniProt ID, in TTDR00444 and TTDR00445 the (same) 11 compounds are mapped to the gamma secretase presenilin 1 and 2 subunits, respectively (P49768, P49810).

Two other important questions are the species split between human vs. non-human and the Swiss-Prot-to-TrEMBL ratio (SP:TR). Results from the UniProt query interface[20] are shown below (Table 6).

**Table 6:** Comparison of species and Swiss-Prot: TrEMBL ratios (SP:TR).

	ChEMBL	DrugBank	HMDB	TTD
All	5797	715	5647	1757
Human	2621	576	5231	1277
Species/strain total	401	63	375	173
Largest non-human	Mouse (698)	*E.Coli* (45)	Hep C (32)	*E.Coli* (89)
SP:TR	5222:575	691:24	5376:271	1620: 137
Human TR-only	20	4	270	12

The details cannot be expanded here but some features can be interpreted. In the case of ChEMBL, as might be expected from the wide range of primary medicinal chemistry literature extracted, the zoo of targets includes 45% human and 15% rodent (698 mice plus 199 rat). Some of the other 400 species seem counter-intuitive as drug targets, such as Q42656 from *Coffea Arabica* (CHEMBL5217) with 94 compounds aligned against it. It turns out this is a mechanistic exemplar enzyme for cross-screening human alpha-mannosidase inhibitors as potential antitumor agents. DrugBank has a distinct human vs. anti-infectives split but is rodent-free because orthologous substitution has been their chosen curatorial practice. This means human protein IDs replace rodent or other mammalian proteins specified in the references for the drug entries.[11] Analogously, there are a number of gram+ve antibacterial compounds where the *E.Coli* orthologue has been added or substituted for the *Staph.* or *Strep*. target. Many species are also included in TTD but TTDR00218 (O23733), a cysteine synthase from *Brassica juncea* is an error. The viral strain polymerases in HMDB will be removed during a current revision of protein mappings.[11]

The topic of TrEMBL entries generated by automated annotation is also too detailed to go into here. However, while the number of human TrEMBL entries is low (Table 6) their presence indicates probable curatorial errors and/or updating lapses. The reason is that the Swiss-Prot expert review process is essentially complete for the human canonical proteome and certainly for all plausible drug targets. Thus, human mappings should have a Swiss-Prot ID rather than a TrEMBL (or both). To be fair, the current human SP:TR ratio of 20 255: 113 824 makes assigning an incorrect (or quasi-duplicate) entry an easy curatorial error to make because of the 5-fold excess of accession numbers for proteins with partially shared automated annotation but related by splice forms, minor sequence differences or as fragments.

**Figure 7:**
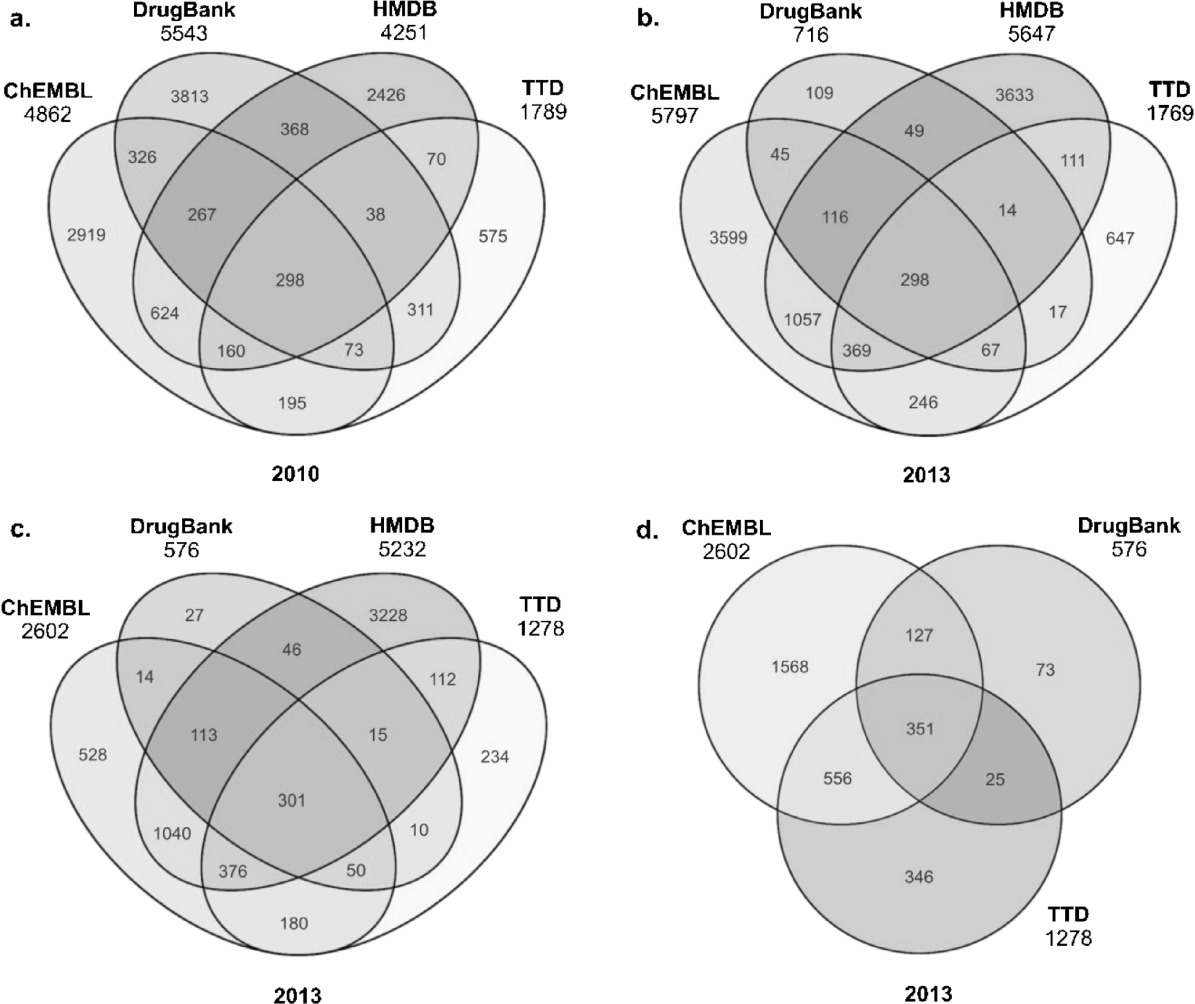
Four-way Venn diagrams of UniProtID content. These are shown as: a) 2010, b) 2013, c) human-only 2013, and d) human-only for the three drug databases in 2013. The ratio of intersect between the sources and the total union in each case is: a) 298:12512, b) 298:10036, c) 301:6275, and d) 351:3046.

The data underline the marked differences in species capture between the four databases. We chose human-only comparisons since they have a number of advantages compared to using total UniProt IDs. Firstly, this normalises the comparison (i.e. apples vs. apples). Secondly, we can compare the three drug-centric databases as a separate set and, thirdly, comparisons of protein function distribution are more valid for single-species. Only salient features can be highlighted, but note that Venny can be used to reproduce any of these via the supplementary data protein lists. Given the caveats mentioned for 2010 vs. 2013 three points are clear from Figure 7a and 7b. Total protein capture is high, the consensus is low and the four databases have, in general, further diverged. Taking human-only reduces the 2013 divergence (Figure 7c). Comparing just the drug databases for human-only increases the 3-way consensus (Figure 7d) to a probable approximation of approved drug targets but the union of the three encompasses over 15% of the genome.

### 4.3 Protein Functional Categories

**Figure 8:**
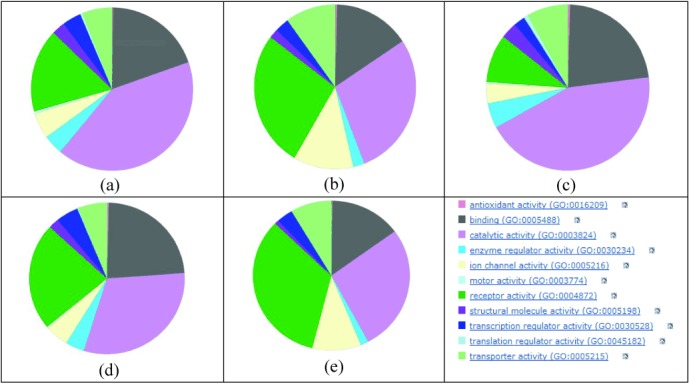
Gene Ontology molecular function distributions. These are shown as top-level categories for human proteins in: (a) ChEMBL, (b) DrugBank, (c) HMBD, (d) TTD, and (e) the three-way consensus of ChEMBL, DrugBank and TTD. The last panel has the colour key to the GO molecular function categories. Total proteins in each case are the central intersects from Figure 7c and 7d.

Having normalised the databases to human protein IDs there are many options for property and annotation comparison that could provide insights into coverage selectivity. We have chosen the Genome Ontology (GO) molecular function because this is conveniently generated via the Panther web site.[21] This usefully provides a functional distribution for any set of protein IDs but with the caveat that GO terms are both nested (i.e. top categories are broad) and forked (some proteins are assigned to multiple functions). While this means the derived pie charts should not be over-interpreted, they are nonetheless useful for a broad-brush comparison of sources (Figure 8).

From left to right, both ChEMBL and HMDB have the highest proportions of enzymes. It is also clear that HMBD has a capture scope that extends beyond metabolic enzymes. DrugBank is similar to TTD but the latter has proportionally less ion channels and receptors. DrugBank looks most similar to the consensus proteins in the high proportion of receptors, enzymes and ion channels. This is unsurprising, since Figure 7d indicates 60% of the primary target proteins are subsumed into the 3-way consensus.

### 4.4 Approved Target Lists

The target proteins of approved small-molecule drugs are of intense interest but listings that have appeared in the literature have not typically been compared. We have used this work as an opportunity to make such comparisons by using three human-only sets. The first of these is the 3-way consensus between the 2013 versions of ChEMBL, DrugBank and TTD (i.e. the 351 proteins in the centre of Figure 7d). The second is a list of targets derived from a re-curation of DrugBank in 2011 (RAS set).[22] The third is an extended list from a commercial database compiled in 2011, each of which had chemical modulation data in papers or patents (SOU set).[23]

**Figure 9:**
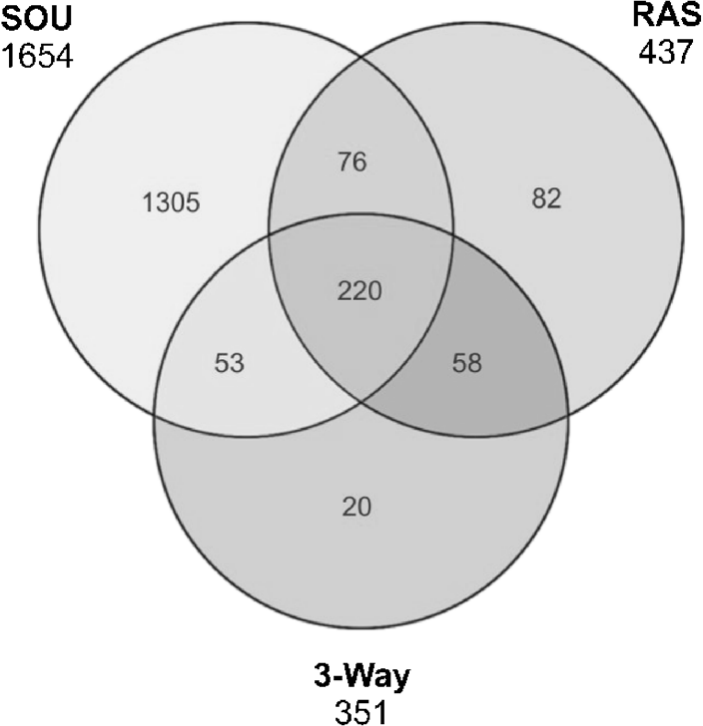
Venn diagram of approved drug targets sets. The SOU 1654 set is from PMID 21569515, RAS 437 from PMID 21804595 and the 3-Way, 351, is the ChEMBL, DrugBank, TTD intersect from Figure 7d.

Notably, we see concordance and discordance. While the common set is only 220 targets it represents a five-way consensus (although the RAS set used an earlier DrugBank version as the starting point the final list was independently curated). Analogously, the overlap sets of size 53, 58 and 76 represent a two-way consensus. The set with at least two intersects (407) would thus be a good approximation to a human primary target set (up to 2011). The extensive unique content from the SOU set is expected because this includes a “long tail” of research targets, 473 of which had a chemistry-to-protein relationship curated from only one document. We can utilise GO classification charts (Figure 9) to compare the protein lists from different sections of the Venn diagrams.

**Figure 10:**
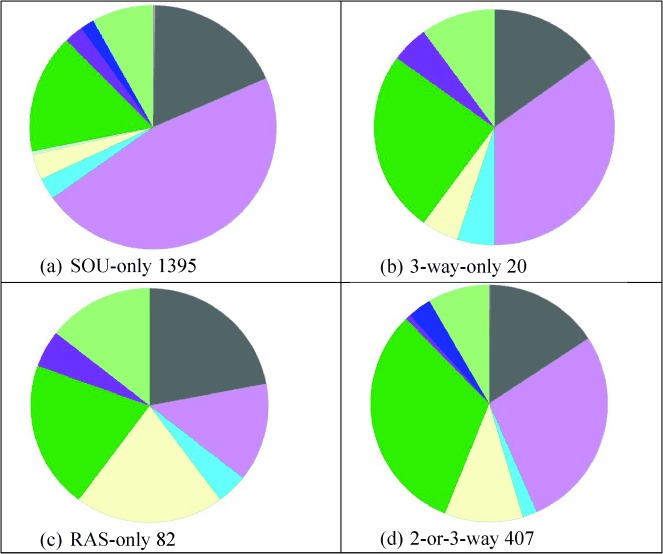
Gene Ontology molecular function distributions for approved target sets.

Taking the 2-or-3-way set first, not unexpectedly, because it contains only 56 more proteins, this looks similar to the 351 protein set from the 3-way drug database consensus and DrugBank primary targets. Also not unexpected is that the large number of proteins in the SOU-only set show the highest proportion of enzymes because these constitute a substantial part of the long tail of research targets. Given that the other two unique sets are small, the distributions need to be interpreted with caution, but they do indicate differences. While the selectivity that might have caused this would need a detailed analysis, we can take one example. The dark purple sector in Figure 10b contains structural proteins (GO: 0005198) that are not typically drug targets. It turns out that one of these unique to the 3-way set is Tubulin beta-2 chain (P68371). This duly has entries in the three databases as TTDS00389, CHEMBL1848 and DrugBank target 2499. However, the large number of tubulin protein components for different species have resulted in inconsistent curatorial choices for microtubule modulators reported in the literature. In the SOU set these may have been assigned to microtubule as a target designation (i.e. without a protein ID) and in the RAS set to Tubulin beta chain (P07437). The small molecule mappings for this mechanism of action are rendered even more complex because vincristine (CHEMBL303560) is mapped to three non-human tubulin proteins in ChEMBL, whereas in DrugBank, the anthelminthic albendazole (DB00518) is ortholgously mapped to two human tubulins (Q71U36 and P68371). Because these are both classified as pharmacologically significant, these proteins are included in the DrugBank human targets for approved drugs.

**Figure 11:**
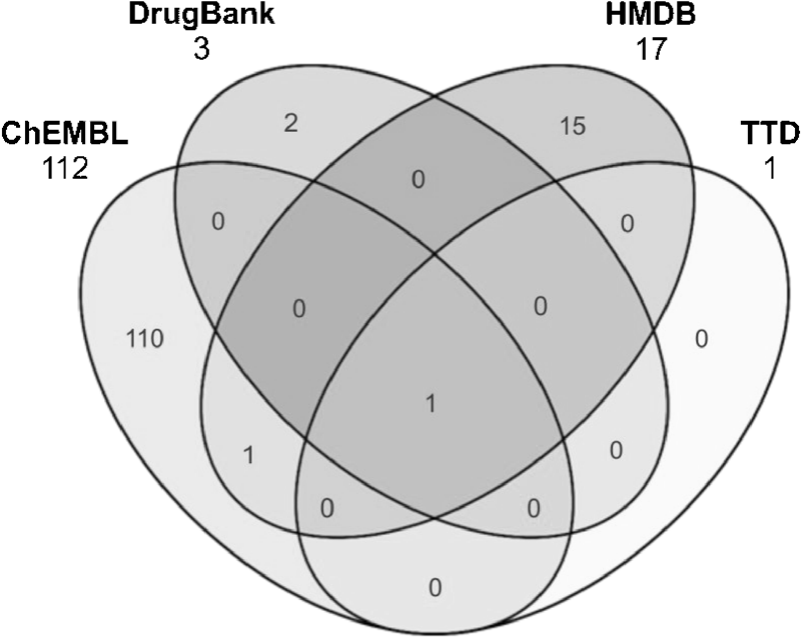
Venn diagram of protein identifiers linked to atorvastatin. Totals are indicated at the top of each ellipse.

Our final protein content comparison looked at mappings associated with just one drug, atorvastatin (Lipitor), an inhibitor of 3-hydroxy-3-methylglutaryl-coenzyme A reductase, HMGCR (P04035). The Venn diagram (Figure 11) shows pronounced differences. It is important to note that these protein relationships are to the parent molecule (i.e. not salts) in each case (CHEMBL1487, DB01076, HMDB05006 and DAP000553). All four have one target (P04035) in common but no others. Notably, ChEMBL connects 112 proteins to atorvastatin in 2013 but only three in 2010. It turns out the majority of new mappings come from a Drugmatrix panel screen added in ChEMBL v.15. This includes 103 proteins with 1742 associated activity measurements (CHEMBL1909046). Compared to the other three databases where the predominant relationship is selected as an activity modulation, panel screens can record the absence of activity (at the maximum concentration tested) across a substantial proportion of the matrix, but mappings are recorded in the database for every protein in the panel (i.e. the relationship is “has been assayed”). In SAR terms the inclusion of both activity and inactivity are important. However, users then need to apply filtration and threshold judgements when querying the data for their particular needs.

Beyond one other protein in common (P08684, Cytochrome P450, 3A4) the remaining unique assignments indicate marked differences in relationship capture between the databases. As can be seen in the Venn diagram, TTD has P04035 mapped to atorvastatin as single primary target. On the other hand, the DrugBank record, (DB01076) is mapped (in 2010 and 2013) to two additional targets, Dipeptidyl peptidase 4 (DPPIV, P27487) and the Aryl hydrocarbon receptor (P35869). Only P04035 is captured in the 2013 download because this is tagged as known pharmacology. It turns out that DPPIV is also linked to atorvastatin in ChEMBL but to pig not human (P22411). Inspection of the record shows this to be the correct link via PMID 18068977 but the data quoted in the abstract (i.e. a *K_i_* of 58 µM) is not captured in the record. DrugBank cites the same reference but orthologously substitutes pig for human and tags the relationship as having no pharmacology. Given the high *K_i_* this seems an appropriate curatorial judgment (this transitive assignment has been incorporated into the PubChem record for atorvastatin). However, further mapping complexity is introduced via CHEMBL393220. This maps the atorvastatin calcium salt as a separate entity to a series of antimalarial whole-parasite screens plus rat HMGCR (P51639). For HMDB three of the 17 proteins have reaction schema related to HmgCoA metabolism. However, the reasons for mapping the other 14 proteins into this entry are unclear.

## 5 Conclusions

Many aspects of databases can be compared in order to discern utility. These include the data model, web interface, query and navigation functionality, cross-links, download sets, API availability, facility for integration, PubMed content and mapped relationship distributions (e.g. proteins-per-compound and compounds-per protein). Despite the impact of these on exploitation we have had to limit ourselves here to what we consider the two most important features of chemistry and protein content. The approaches outlined are generic and we thus encourage others to perform such studies, especially where broader adoption of InChIKey and UniProtIDs make comparisons more straightforward than hitherto. The databases in this study provided useful download options but differ in the extent of post-download processing necessary for standardised comparisons. Thus, broader intra-database harmonisation would be welcome, for example, if each of the chemical and target sets could be downloaded as SD files, protein ID lists in Excel, UniProt cross-references, have an API (as ChEMBL has already) as well as complete and selectable PubChem sets.

Our chemistry results show that all the sources have improved. This is likely to be a combination of enhanced structure handling rules and manual curation. While this has increased overlap there is also divergence and expansion. This enhances their complementarity and aggregate coverage. Nevertheless, questions as to “how they became the way they are” are important for exploitation. In this respect the ChEMBL publications, regular release notes, and explanations of their medicinal chemistry journal triage, go a long way to answering these questions. While the other databases are also well described in their publications, analysis is necessary to discern selectivity that is not made explicit. Examples include the fact that DrugBank is still over 50% PDB derived, TTD has used ChEMBL for their expansion, HMDB contains many drugs and that lipids are now a major proportion of HMBD content.

A limitation of the current study is the restriction to exact matches between the chemistry collections. Apparent increases in divergence by this parameter may not necessarily translate into wider chemical coverage. In this respect, the impact of these databases is not only on accessing data, but using it to develop models. Thus, updates will have relatively little impact on QSAR and similarity models if similarity remains high. Further work would be needed to see if distribution of pairwise similarities (2D and/or 3D) had shifted significantly between 2010 and 2013.

The aggregate protein content for the four databases in 2013 shows a major expansion encompassing not only binding events in the thermodynamic sense but also biochemical and pharmacological activity. However, the high numbers we have recorded, along with some of the individual examples, indicate divergent curatorial rules and stringencies for each source. For example, the three drug-centric databases (Figure 7d) cover 3046 human proteins (i.e. ∼15% of the genome). This contrasts with the active compound mapping from an extensive corpus of literature and patents that recorded relationships to 1654 proteins in 2011, although a subsequent report added internal company data to support up to 2000 mappings.[23–24] The 5232 proteins in HMDB also provide a maximal-mapping example, since a different approach recorded only 1653 human metabolic enzymes.[25] There are arguments for extending mappings to indirect and assayed relationships, as opposed to a more stringent restriction to only potent activity modulation or direct metabolic interaction. Nevertheless, extended mappings can be problematic. For example, they have the potential to confound Linked data integration if the source-specific filtration options are submerged.[26] A second concern is proliferation beyond the sources they were first incorporated in. This can occur not only via automated cross-linking but also by curatorial transfer between databases.

## References

[1] Southan C, Varkonyi P, Muresan S (2009). J. Cheminf.

[2] Gaulton A, Bellis LJ, Bento AP, Chambers J, Davies M, Hersey A, Light Y, McGlinchey S, Michalovich D, Al-Lazikani B, Overington JP (2012). Nucl. Acids Res.

[3] Knox C, Law V, Jewison T, Liu P, Ly S, Frolkis A, Pon A, Banco K, Mak C, Neveu V, Djoumbou Y, Eisner R, Guo AC, Wishart DS (2011). Nucl. Acids Res.

[4] Wishart DS, Jewison T, Guo AC, Wilson M, Knox C, Liu Y, Djoumbou Y, Mandal R, Aziat F, Dong E, Bouatra S, Sinelnikov I, Arndt D, Xia J, Liu P, Yallou F, Bjorndahl T, Perez-Pineiro R, Eisner R, Allen F, Neveu V, Greiner R, Scalbert A (2012). Nucl. Acids Res.

[5] Zhu F, Shi Z, Qin C, Tao L, Liu X, Xu F, Zhang L, Song Y, Liu X, Zhang J, Han B, Zhang P, Chen Y (2012). Nucl. Acids Res.

[6] Muresan S, Sitzmann M, Southan C, Larson RS (2012). Bioinformatics and Drug Discovery.

[7] Gregori-Puigjané E, Garriga-Sust R, Mestres J (2011). J. Comp. Chem.

[8] Ihlenfeldt WD, Takahashi Y, Abe H, Sasaki S, Chem J, GmbH Xemistry (1994). Inf. Comp. Sci. http://xemistry.com/..

[9] Sitzmann M, Filippov IV, Nicklaus MC (2008). SAR QSAR Environ. Res.

[10] Heller S, McNaught A, Stein S, Tchekhovskoi D, Pletnev I (2013). J. Cheminfo.

[12] http://bidd.nus.edu.sg/group/cjttd/TTD_download.txt.

[13] http://www.drugbank.ca/system/downloads/current/target_links.csv.zip.

[14] http://www.uniprot.org/uniprot/?query=database%3A%28type%3Achembl%29&sort=score.

[15] Oliveros JC (2007). VENNY. An interactive tool for comparing lists with Venn diagrams. http://bioinfogp.cnb.csic.es/tools/venny/index.html.

[16] Tyrchan C, Blomberg N, Engkvist O, Kogej T, Muresan S (2009). Bioorg. Med. Chem. Lett.

[17] Geer LY, Marchler-Bauer A, Geer RC, Han L, He J, He S, Liu C, Shi W, Bryant SH (2010). Nucl. Acids Res.

[18] Wein SP, Côté RG, Dumousseau M, Reisinger F, Hermjakob H, Vizcaíno JA (2012). Nucl. Acids Res.

[19] Mi H, Lazareva-Ulitsky B, Loo R, Kejariwal A, Vandergriff J, Rabkin S, Guo N, Muruganujan A, Doremieux O, Campbell MJ, Kitano H, Thomas PD (2005). Nucl. Acids Res.

[20] http://www.uniprot.org/docs/dbxref.

[21] Mi H, Muruganujan A, Thomas PD (2013). Nucl. Acids Res.

[22] Rask-Andersen M, Almén MS, Schiöth HB (2011). Nat. Rev. Drug Discov.

[23] Southan C, Boppana K, Jagarlapudi S, Muresan S (2011). J. Cheminf.

[24] Eriksson M, Nilsson I, Kogej T, Southan C, Johansson M, Tyrchan C, Muresan S, Blomberg N, Bjäreland M (2012). Mol. Inf.

[25] Romero P, Wagg J, Green M, Kaiser D, Krummenacker M, Karp P (2004). Genome Biol.

[26] Samwald M, Jentzsch A, Bouton C, Kallesoe C, Willighagen E, Hajagos J, Marshall M, Prud’hommeaux E, Hassanzadeh O, Pichler E, Stephens S (2011). J. Cheminf.

